# The Geography of Women’s Empowerment in West Africa

**DOI:** 10.1007/s40980-021-00099-2

**Published:** 2023-01-21

**Authors:** Jacqueline Banks, Stuart Sweeney, Wendy Meiring

**Affiliations:** 1 Minnesota Population Center, University of Minnesota, Minneapolis, MN 55455, USA; 2 Department of Geography, University of California, Santa Barbara, CA 93106-2150, USA; 3 Department of Statistics and Applied Probability, University of California, Santa Barbara, CA 93106-3110, USA

**Keywords:** West Africa, Women’s empowerment, Bayesian multinomial structured geo-additive regression

## Abstract

Women’s empowerment has been a subject of interest because of its relevance to development and demography, particularly in West Africa. Women’s empowerment is typically conceptualized as an individual attribute of women, associated with socioeconomic and demographic characteristics. However, we hypothesize a geography of women’s empowerment in the West African region, where empowerment processes are culturally situated and embedded in place. Such a geography would be observable via spatial associations over the region. This study uses Demographic and Health Survey data from 14 West African states over the past decade and an innovative multi-stage approach combining advanced statistical methods and spatial assessment to analyze indicators of women’s empowerment and its spatial variability across the West African region. First we use a multivariate classification method to identify patterns in responses to empowerment questions and derive an empowerment classification scheme. Next we use these classifications to render a map of West Africa depicting the spatial variation of women’s empowerment in the region. Ultimately, we fit multinomial structured geo-additive regression models to the data to analyze spatial variation in women’s empowerment while controlling for certain socioeconomic-demographic characteristics. Our results demonstrate that women’s responses to empowerment survey questions indeed vary geographically, even when controlling for individual socioeconomic-demographic attributes. This finding suggests that women’s empowerment may relate to aspects of culture embedded in place in addition to the ways it relates to socioeconomic and demographic characteristics.

## 1 Background

In the past half century there has been immense international interest in the development of West Africa, often with the putative goals of women’s empowerment and gender equality. The International Women’s Conference in Mexico City in 1975 declared it the International Year of Women and the beginning of the International Decade for Women (Kandeh & Kannan, 2014). From the third UN Women’s Conference in Nairobi in 1985, which focused on placing gender at the center of development, came The Nairobi Forward-Looking Strategies for the Advancement of Women which proposed a handful of specific goals for development (ibid). The United Nations Conference on Population and Development (ICPD) in Cairo in 1994 marked a paradigm shift in the international population community where women’s empowerment replaced neo-Malthusian concerns of overpopulation in international development and population policy (Presser & Sen, 2000; Hodgson & Watkins, 1997). Gender equality and women’s empowerment was named by the United Nations as one of eight Millenium Development Goals in 2000 and one of the 17 Sustainable Development Goals of 2015 (United Nations, 2000, 2015). Women’s empowerment has persisted as an international value because, in addition to its intrinsic value, it is widely thought to be associated with slower population growth and important for development, particularly in low- and middle-income countries with high fertility rates like those in West Africa (Sasser, 2018; Halfon, 2007; Hartmann, 2016). Empirical research has documented association between women’s empowerment and women’s fertility preferences, family planning, contraceptive use, and fertility outcomes (Upadhyay et al., 2014; Atake & Gnakou Ali, 2019; Prata et al., 2017). Consequently, since the 1994 ICPD, sub-Saharan African countries have adopted significantly more population policies related to norms about women’s rights because of their international ties with non-governmental organizations (Robinson, 2015).

Despite the global consensus supporting the importance and value of women’s empowerment, there remain inconsistencies in the definition and opertionalization of women’s empowerment (James-Hawkins et al., 2016; Malhotra & Schuler, 2005; Yount et al., 2016). Empowerment, though often conflated with women’s status, education, employment or gender inequality is theoretically distinct. Conceptual clarity in this regard is provided by a few seminal works providing working definitions of empowerment. Most notable are those of Kabeer (1999, 2005) in which she describes empowerment as a process by which women gain agency, where agency is defined as the ability to make meaningful life choices. This framework, which centers agency as a core, defining component of empowerment is very commonly cited in the literature in this domain (Malhotra & Schuler, 2005; Upadhyay et al., 2014; Prata et al., 2017; Samari, 2017, 2019a; Asaolu et al., 2018; Yount et al., 2016; James-Hawkins et al., 2016; Lee-Rife, 2010; Heckert and Fabic, 2013) and has been further developed and refined since (Richardson, 2018). Under this framework resources are necessary and non-sufficient pre-conditions of empowerment (Kabeer, 1999) and the medium through which agency is exercised (Kabeer, 2005). Agency is the process undergirding empowerment, and the realized outcome of empowerment are achievements (Kabeer, 2005; Richardson, 2018). Resources include conventional, material resources as well as various human and social resources (Kabeer, 1999). This could include education, wealth, employment, certain social relations, or access to means.

The challenge in basing an operationalization of empowerment on this definitional framework using survey data derives from the limitations posed by cross-sectional observation in measuring a process. Kishor (2000) offers an alternative conceptual framework which is amenable in the application of analysis of demographic and health survey data, while maintaining the conceptualization of empowerment as a process. This framework outlines three distinct stages of the empowerment process, separating “settings” and “sources” as precursors of empowerment and “evidence” as the outcome of empowerment. Observation of all three components provides a snapshot of empowerment on a population level in cross sectional data (Kishor, 2000; Kishor & Gupta, 2004; Kishor & Subaiya, 2008). The “evidence” described in Kishor’s framework can be analogous to Kabeer’s “achievements” or “agency” depending on whether it is agency or something else that is the desired outcome.

One key difference between these frameworks is that Kishor’s “setting” for empowerment refers to the circumstances of women’s lives which are or are not conducive to empowerment, an aspect which is not explicit in Kabeer’s “resources”, though one could interpret resources to include such circumstances. “Setting” as a distinct precursor alludes to the importance of the women’s social and cultural environment, which may be aptly described with the geographic concept of “place”. Place refers to the rich embodiment of culture in a location, which accumulates as a historical process of interaction between humans and the natural environment. We theorize that women’s social and cultural environment is an important component of women’s empowerment and functions as part of the set of preconditions of empowerment. Social and cultural norms are implicated in the meaning of places and include *injunctive norms* (relating to beliefs about what one should or should not do) and *descriptive norms* (which are reinforced by observed and practiced behaviors) (Shakya et al., 2019). Though agency as a component of empowerment is conceptually distinct from setting, the manifestation of intrinsic agency (relating to beliefs and attitudes) and instrumental agency (relating to effective actions) (Miedema et al., 2018; Samari, 2019b) in a place would in turn affect injunctive and descriptive norms respectively in that place and would then contribute to that setting and the making of that place. The social and cultural settings that constitute places are elusive, however geographic space and location serve as functional proxies for places. We expect that measurable components of empowerment, like agency, are observable via geographic patterns of women’s empowerment data.

The process of empowerment as articulated by Kabeer is only meaningful in the context of disempowerment, where women have been denied choice (Kabeer, 1999). This subordination of women is ubiquitous in patriarchal societies across the world, yet the specific set of beliefs, practices, and institutions through which gendered power dynamics persist or change are culturally situated and embedded in geographic places. What is unique about the status of women in West Africa in relation to other parts of the world is that women’s subordinate status is intensified by a combination of national underdevelopment, poverty, food instability, a long history of local patriarchal cultures, a shorter history of European colonization with its own patriarchal culture, post-independence political instability, high fertility rates, urbanization and westernization. A study of the cultural attitudes toward women and of women’s behavior in West Africa is challenging not only because of the region’s ethnic and religious diversity but also because of the region’s complex cultural geography. Historically, ethnic groups have resided in specific regions which do not correspond with the boundaries of modern nations. Consequently the richness of this complex cultural geography is not properly depicted via national or cross-country comparison of cultural markers.

In conceptualizing women’s empowerment as a complex, multidimentional, and culturally-situated phenomenon, we theorize that women’s empowerment—and concomitant ideas, attitudes, and practices—would spread spatially through cultural diffusion and social interaction processes whereby individuals and communities spread information, ideas, and behaviors and influence one another (Bongaarts, 2006). It was through these diffusion and social interaction processes that fertility transition, and the uptake of family planning practices, spread through Europe during its development in the period 1870–1960 (Bongaarts, 2006; National Research Council, 2001). The famous result from the work of Ansley Coale in the 1970’s on Europe’s transition showed that socioeconomic conditions were only weakly predictive and that this geographic patterning was more relevant in understanding fertility transitions (Watkins, 1987). Similarly, recent research demonstrates cultural diffusion of norms via social networks which impact fertility behaviors in low-income populations as well, and are observed in spatial analyses of fertility and fertility-related cultural norms (Shakya et al., 2019). The present-day West African region is situated in a highly complex, globalized modern world where even individuals in remote places may be potentially exposed to influence from cultures around the world. Exogenous ideas, practices, or values can be introduced to West African people via direct interest in women’s empowerment like population policies promoted by non-governmental organizations (Robinson, 2015; Schnable et al., 2020), population health interventions or programs (Olivier de Sardan et al., 2017) or even through data collection and government demographic surveillance (Chatterjee & Riley, 2018; Adams, 2016). However these influences are not automatic and individuals are not equally and universally exposed to these influences nor adopting them. Rather these ideas, attitudes, and practices take root through interactions between proximate individuals which aggregate spatially at higher levels (community, village, regional etc).

The importance of geographic context and cultural specificity in understanding women’s empowerment and empowerment metrics has been raised by some scholars (Kazembe, 2020; James-Hawkins et al., 2016). Yet there remains a scarcity of studies which specifically analyze geographic patterning of women’s empowerment or examine the role that geography plays in how women respond to questions used for empowerment metrics (see Kishor & Gupta, 2004 for one such study). There are a few studies that support the notion of a geography of empowerment in sub-Saharan Africa. Sarkar et al. (2016) documented heterogenous improvements in girls’ secondary education and wage employment at subnational levels that are obscured in country-level analyses. In another geographical analysis of gender inequality at subnational levels based on a comparison of male and female headed households’ wealth, Fisher and Naidoo (2016) identify spatial heterogeneity with bands of high and low gender equality in West Africa. Kazembe (2020) identifies a geography of empowerment in the country of Namibia with spatial patterning of multiple dimensions of empowerment.

It is important to note that women’s empowerment is considered to be a complex and multidimensional concept where dimensions of empowerment are distinct and possibly not correlated (Richardson 2018; Malhotra & Schuler, 2005; Mason, 1986). Most recent studies of empowerment aim to incorporate this multidimensionality through the use of multiple indicators of empowerment as composites/sums, indices, or through factor analysis (Upadhyay et al., 2014; Prata et al., 2017). Certain studies have used these multivariate methods to validate multidimensional measures of empowerment in certain geographic contexts like those of Ewerling et al. (2017), Asaolu et al. (2018), and Miedema et al. (2018) in sub-Saharan Africa and Yount et al. (2016) and Samari (2019a) in Egypt. However, very few studies exist which analyze multiple dimensions of empowerment spatially (Kazembe 2020).

On the surface, our aim in this study is to describe how women’s empowerment manifests and varies geographically across the West African region. We do this by identifying spatial patterns of agency—a core component of empowerment—and patterns that are not attributable solely to individual socio-demographic characteristics and may illuminate places which are settings of empowerment. In doing so we hope to create a notion of a geography of empowerment. This notion offers scholars and policymakers a consideration of the importance of the socio-cultural environment in which individual women’s lives are situated in addition to socio-demographic characteristics of individuals. This would contribute a geographic perspective to the fields of demography and development in better understanding women’s empowerment in low- and middle-income countries.

## 2 Data

The data used in this study come from The Demographic and Health Surveys (DHS) Program which conducts surveys across the developing world in collaboration with participating countries and is funded largely by the United States Agency for International Development (USAID). We use data from 19 nationally representative surveys conducted in the prior decade in fourteen West African states: Benin (2011–12, 2017–2018), Burkina Faso (2010), Cameroon (2011), Cote D’Ivoire (2011–12), The Gambia (2013), Ghana (2014), Guinea (2012), Liberia (2013), Mali (2012–13), Niger (2012), Nigeria (2013), Senegal (2017, 2016, 2015, 2014), Sierra Leone (2013), and Togo (2013–14). These surveys have large sample sizes ranging from 5000 to 30,000 households. The DHS uses a stratified, two-stage probability sample design where within each stratum a primary sampling unit (PSU) is selected, and then households are randomly selected from within each PSU. Women from each household answer a long questionnaire that includes information regarding health, wealth, and demographics.

There are several sets of questions in the survey that are of particular interest in this study because they were developed based on the aforementioned framework for operationalizing empowerment first articulated by Kishor (2000) and explicitly intended to indicate empowerment cross-nationally (Kishor & Subaiya, 2008). These include questions about household decision-making, attitudes towards domestic violence, experience of domestic violence, control issues in a relationship, earnings and how they are spent, and home and land ownership. However, due to limitations of the multivariate methods used in our study which bar data sets with missing values we excluded variables which were not asked in every country-survey or which had many missing values. The full sample, 234,620 respondents, is reduced to the subset of those with responses to each of the included questions, 145,971 respondents. These data are not missing at random, questions about household decision-making were only asked of women in relationships.

The first set of questions included in our analysis regards household decision-making. These questions are the most commonly used indicators of empowerment, particularly when studying empowerment in relation to women’s fertility and family planning (Upadhyay et al., 2014; Prata et al., 2017; Richardson, 2018). They are considered to be “instrumental” measures of agency (Samari, 2019a, b; Miedema et al., 2018) and constitute a direct proxy of agency in Kabeer’s framework (Malhotra and Schuler 2005) and evidence of empowerment in Kishor’s framework (Kishor and Subaiya, 2008). Women were asked who usually makes decisions about: (a) her own health care, (b) large household purchases, (c) visits to family or relatives, and (d) what to do with money her husband earns. Possible responses for each can be any of the following: (1) the respondent makes the decision alone, (2) her and her husband make the decision together, (3) their husband makes the decision alone, or (4) someone else makes that decision. In the question regarding the money husbands earn, they can also respond that their husband has no earnings.

The second set of questions we include has to do with attitudes towards domestic violence. These metrics are also justified indicators of empowerment in the literature (Upadhyay et al., 2014; Prata et al., 2017). They are commonly used measures of gendered attitudes and beliefs (Richardson, 2018) and are considered to be “intrinsic” measures (Samari, 2019a, b; Miedema et al., 2018) and are of importance in sub-Saharan Africa (Asaolu et al., 2018). Women are asked if, in their opinion, a husband is justified in hitting or beating his wife in each of five proposed situations: (a) if she goes out without telling him, (b) if she neglects the children, (c) if she argues with him, (d) if she refuses to have sex with him, and (e) if she burns the food. For each of these five questions the respondent can say either “yes”, “no” or “don’t know”.

The final question included in our analysis asked women when they believed women are justified in refusing to have sex with their husbands. This indicator of gendered attitudes is appropriate for the study of women’s empowerment in sub-Saharan Africa (Heckert & Fabic, 2013). In the standard questionnaire this question asks “Is a wife justified in refusing to have sex with her husband when she knows he has sex with other women?” to which the woman can respond with either “yes”, “no” or “don’t know”. Inclusion of these three dimensions of women’s agency—household decision-making, justifications of violence, and justifications to refuse sex—provides the means to construct a multidimensional indicator of agency, a core component of empowerment, that is validated for cross-cultural and geographic comparison (Asaolu et al., 2018; Ewerling et al., 2017; Kazembe, 2020; Heckert & Fabic, 2013).

Each observation, meaning the responses for each woman interviewed, is associated with a PSU, and each PSU has a unique associated geographic coordinate. The number of women that share a set of coordinates, i.e., the number of women interviewed within each PSU, ranges from roughly 15–100 women. To protect participant anonymity, the geographic coordinates provided by DHS are not exact locations but are instead randomly offset by up to two kilometers in urban regions and 5 km in rural regions. The random error imposed on the coordinates does not pose a problem for our analysis or interpretation since the geographic scope of our study is very large—the entire West African region—and we are interested in broad patterns of coherence in the spatial pattern. Furthermore, the data positions PSU’s within first-level administrative regions within countries. Note that geographic coordinate data are not collected in The Gambia survey and are collected but not distributed for the surveys in Niger. However, using data about the first-level administrative regions we are able to maintain these countries in all steps of the analysis that do not require precise locations.

## 3 Methods

Our study design is composed of three stages of analysis. The first stage uses multivariate statistical cluster analysis on the empowerment data, in order to identify patterns in responses to empowerment questions. This classifies women into empowerment “clusters” which we use to indicate categories of type or level of empowerment. In the second stage, we construct a map of the West African region depicting the resulting empowerment categories and do a simple visual analysis of geographic patterns. In the third stage we use a statistical model to describe variation in an individual’s membership in an empowerment category, in terms of socioeconomic-demographic characteristics and geographic location. The model used is a multinomial structured geo-additive regression (STAR) model which supports controlling for socio-demographic factors and clustered DHS sampling scheme, in order to investigate presence of an additional spatially-structured effect.

Cluster analysis is a class of methods that are used to categorize observations into one of several natural groupings or “clusters”; it differs from several other multivariate classification methods in that it relaxes assumptions about the group structure (Johnson & Wichern, 2007). Clustering in general does not result in optimal solutions across all loss functions because the choice of clustering criteria is subjective and there are many clustering algorithms that use different criteria, have different advantages or disadvantages, and will produce different results. All clustering algorithms are based on calculations of “distance” from an observation to the clusters and then observations are assigned to the cluster that minimizes that distance. For this reason, clustering algorithms can be computationally costly because they often involve creating a large matrix of pairwise distances between observations. Because our empowerment data are all nominal categorical variables, options for clustering algorithms and distance metrics available are limited. In our analysis we chose to use a clustering algorithm called k-modes. K-modes is a non-hierarchical method developed by Huang (1998) and is an adaptation of the better-known k-means developed by MacQueen (1967); (Johnson & Wichern, 2007). The main advantages of using the k-modes algorithm is that it is intended for use with nominal data and it is computationally fast because it does not require a distance matrix. This algorithm works by comparing the “distance” of each observation to each of k different “modes” using a simple matching distance, counting the number of mismatches in all variables between an observation and a mode. We chose this algorithm for computational efficiency, although we do recognize that it has several limitations, including that it requires a predetermined, fixed number of clusters, k. Compared to several hierarchical algorithms, it may be difficult to control the size of clusters in k-modes, and the algorithm treats missing values as another nominal level which is less than ideal. Lastly, the algorithm requires specification of k unique initial “modes” which can either be predetermined by the user or can be randomly selected from observations in the data.

We exploited this last aspect of the algorithm by priming it with five initial modes that are conceptually meaningful (see Table 1 for the values of the initial modes). The first corresponds to answers we would expect from a woman who is “more empowered”, who says she makes decisions together with her partner, who does not support any of the justifications of violence, and who believes a woman is justified to refuse sex if her partner has other women. The second represents a “less empowered” woman who provides opposite responses from initial mode 1. The third represents what we call the “modal empowered” woman, this is constructed from the most frequent responses to each question: “no” for all justifications of violence, “partner” for all household decision questions, and “yes” for justification to refuse sex. The fourth represents a very “autonomous” woman with responses similar to initial mode 1, but who makes decisions on her own instead of with a partner. The fifth is a residual category “other” comprised of all the neutral responses: “don’t know” for the justifications of violence and refusal of sex questions and “other” for the household decision-making questions. As the algorithm iterates through each observation and categorizes each respondent into one of the k = 5 modes, these modes may change so while the initial modes may prime the categories to be meaningful, the resulting classification need not necessarily match these initial constructs.

After assigning respondents to empowerment classifications, we use this information and the geographic data provided by the DHS to construct a map of the West African region to describe the spatial distribution of empowerment and enable a simple visual assessment. Latitude and longitude coordinates of each DHS PSU are represented as a point on the map and the color of that point represents the relative share of respondents from that PSU that are assigned to each of the three most frequent empowerment classifications. In our k-modes cluster analysis modes 1, 2, and 3 remained unchanged during the clustering analysis and also correspond to the three most frequent empowerment categories. Therefore, we refer to each of the corresponding categories by their conceptually meaningful descriptions. Using blue to represent empowerment category 1 (“more empowered”), red to represent empowerment category 2 (“less empowered”), and green to represent empowerment category 3 (“modal empowered”) on the map, these three colors are blended at each PSU to convey the relative proportions of respondents from this PSU that were assigned to each empowerment category. In this way, the map conveys a dense amount of information easily in one image.

As the final stage of analysis, we fit a multinomial structured geo-additive regression (STAR) model to the data to examine the association between geography and empowerment while controlling for demographic covariates (Fahrmeir & Lang, 2001; Kammann & Wand, 2003; Fahrmeir et al., 2004) . In this model, for each respondent, the response variable is their assigned empowerment category; the geographic/spatial explanatory variable of interest is represented as first level administrative region within countries, and control variables consist of respondent’s age, education level, household income quintile, and an indicator of rural/urban residence. We analyze only the subset of respondents assigned to one of the three dominant empowerment classifications. Using the DHS supplemental data we associate each PSU with a first level administrative region and use these regions to indicate geographic location in the model. Due to the hierarchical survey sampling scheme, our model contains an effect for each PSU; multiple women belong to each PSU, and multiple PSUs belong to each first level administrative region. The appeal of using a STAR model is that it very flexible: It can be used to model non-linear effects, spatial effects, and allows extension for modeling nominal, categorical responses (Umlauf et al., 2015). We model each individual’s empowerment classification as a 3-category multinomial distribution. Model parameters are estimated using Markov Chain Monte Carlo (MCMC) simulation techniques. The model is fit using the R package *R2BayesX* version 1.1-1, which is an R interface to BayesX software (Umlauf et al., 2015; Belitz et al., 2017).

The model specifications follow a similar problem set forth by Kazembe and Namangale (2007), expanded to include the clustered DHS sampling. Let *j* ∈ {1, …, *J*} represent region and *i* ∈ {1, …, *n_j_*} represent the *i*th respondent within region *j*, and suppose this respondent is in PSU *u*(*ij*). Let $Y_{ij}={\left(Y_{ij1},Y_{ij2},Y_{ij3}\right)}^{\prime }$ be a random vector where, for each *k* ∈ {1, 2, 3},

$$Y_{ijk}=\{\begin{array}{cc} 1, & \text{if respondent}\;i\;\text{from region}\;j\;\text{is assigned to empowerment category}\;k\\ 0, & \text{otherwise} \end{array}$$

with empowerment categories as defined earlier. Specifically *k* = 1 represents “more empowered”, *k* = 2 represents “less empowered”, and *k* = 3 represents “modal empowered” category. Let *π_ijk_* equal the probability that *Y_ijk_* = 1. We only include respondents who are classified into one of these dominant three categories in our remaining analyses. This reduction in focus is because the vast majority of women fall into these three categories, and because of practical considerations such as stability of model estimation.

Therefore in our models $\sum_{k=1}^{3}\;\pi_{ijk}=1$ for each *i*, *j*. We assume that

$$Y_{ij}∼\mathrm{Multinomial}(1,\pi_{ij}),\;\text{where}\;\pi_{ij}={\left(\pi_{ij1},\pi_{ij2},\pi_{ij3}\right)}^{\prime }.$$

We set the modal empowered category (*k* = 3) as the reference level within a multinomial logit STAR model. To specify *k* = 3 as the reference level, we set *η*_*ij*3_ = 0 by defining

(1) $$\eta_{ijk}=\ln\;\left(\frac{\pi_{ijk}}{\pi_{ij3}}\right)\;\forall k\in \{1,2,3\}.$$

The components of subsequent models fitted independently to *η_ijk_* for each of *k* = 1 and *k* = 2, are then interpreted as differences in associations relative to the “modal empowerment” reference level. Multinomial probabilities are obtained from (1) via

$$\pi_{ijk}=\{\begin{array}{cc} \frac{\exp\left(\eta_{ijk}\right)}{1+\sum_{\ell =1}^{2}\exp\left(\eta_{ij\ell }\right)}, & \text{if}\;k=1,2\\ 1-\sum_{\ell =1}^{2}\pi_{ij\ell } & \text{if}\;k=3 \end{array}$$

Our models adjust for explanatory variables that are expected to be associated with women’s empowerment in the literature base (Kishor & Subaiya, 2008; Upadhyay et al., 2014; Prata et al., 2017). Let *a_ij_* be age of respondent *i* in region *j*, and ***x***_*ij*_ be a row vector specifying the rural/urban, education and income categories for this individual. Specifically let ***x***_*ij*_[*c*] be the *c*th element in vector ***x***_*ij*_, with ***x***_*ij*_[1] = 1, and other elements given in Table 2. “Secondary+” indicates “secondary or higher” education.

Model (2) is the primary model we use to assess the presence of geographic features in women’s empowerment, while accounting for age and key demographic indicators. The equation for each empowerment cluster *k* ∈ {1, 2} is interpreted relative to the modal empowerment reference category:

(2) $$\eta_{ijk}=x_{ij}\beta_{k}+f_{k}\left(a_{ij}\right)+b_{u(ij),k}+s_{jk}\;\text{for each}\;k\in \{1,2\}.$$

Here *f_k_*(*a_ij_*) is a non-linear p-spline to model association with age for empowerment category *k*, specified as a Bayesian P-spline of degree 3, using 20 equidistant knots (Lang & Brezger, 2004). Vectors ***β***_1_ and ***β***_2_ are each length 8 coefficient vectors for the demographic indicators, with the first element an intercept for that category. For each *k* ∈ {1, 2}, term *b*_*u*(*ij*),*k*_ accounts for the clustered DHS sampling design, modeled as a spatially unstructured effect common to all individuals in empowerment category *k* who belong to the PSU to which respondent *i* within region *j* belongs. Each individual is classified into only one empowerment category, but other individuals within the same PSU may be categorized into different empowerment categories, requiring different PSU effects for each of *k* ∈ {1, 2}. Let *s_jk_* be a spatially dependent (spatially structured) effect for empowerment category *k* in region *j*. Since we use a fully Bayesian analysis, all effects are random.

For each *c* ∈ {2, …, 8} we interpret exp(***β***_*k*_[*c*]) as the relative odds ratio of $\frac{\pi_{ijk}}{\pi_{ij3}}$ with respect to the reference categories: residence = “rural”, education = “none” and income = “poorest”. This statistic is similar to an odds ratio but adapted for the multinomial case (Kazembe & Namangale, 2007). Within a fully Bayesian analysis, we use the default priors, neighborhood definition, and hyperparameter settings in R2BayesX (Umlauf et al., 2015, Belitz et al., 2017). Additional details on priors are in Fahrmeir et al. (2004). Specifically, for each *k* ∈ {1, 2}, we used priors:

*p*(***β***_*k*_) ∝ 1, a diffuse improper prior in 8-dimensions.

The continuous function of age, evaluated at age *a*, can be written in terms of *M* = 23 (number of knots + order) B-spline basis functions as $f_{k}(a)=\sum_{m=1}^{M}\;\gamma_{km}B_{m}(a)$. The prior is a second order random walk on the coefficients {*γ*_*k*1_, ⋯, *γ_kM_*}, given by

$$\gamma_{km}=2\gamma_{k,m-1}-\gamma_{k,m-2}+\epsilon_{km},\;\text{initialized by priors}\;p\left(\gamma_{k1}\right)\;\text{and}\;p\left(\gamma_{k2}\right)\;\propto \;1,\;\text{where}\;\epsilon_{km}\overset{iid}{∼}N\left(0,\tau_{k,a}^{2}\right).$$

$b_{u(ij),k}\overset{iid}{∼}N\left(0,\tau_{k,u}^{2}\right)$ for all PSUs *u*(*ij*)

*s_jk_* has a Markov Random Field prior, specified by conditional distributions of the form

$$s_{jk}|s_{j^{\prime }k}\;\forall j^{\prime }\neq j,\tau_{k,s}^{2}∼N\left(\frac{1}{N_{j}}\sum_{j^{\prime }\in N(j)}s_{j^{\prime }k},\frac{\tau_{k,s}^{2}}{N_{j}}\right)$$

where N(j) is the set of all regions that are neighbors of region *j*, of cardinality *N_j_*. Two regions are neighbors if they share a common geographic boundary.

$\tau_{k,u}^{2},\tau_{k,a}^{2},\tau_{k,s}^{2}\overset{\mathrm{indep}}{∼}IG(0.001,0.001)$, where *IG* stands for Inverse Gamma Distribution.

Priors are independent across *k* = 1, 2. Convergence was diagnosed using MCMC diagnostics discussed further in Sect. 4.

To assess the presence of spatial dependence in women’s empowerment, after adjusting for key covariates and clustered DHS sampling, we compare model (2) to model (3), identical except without the spatial structured component.

(3) $$\eta_{ijk}=x_{ij}\beta_{k}+f_{k}\left(a_{ij}\right)+b_{u(ij),k}$$

For simplicity we call model (2) the “Full Model”, and (3) the “Null Model”. Comparison is made via the deviance information criterion (DIC, Spiegelhalter et al., 2002) and by assessing reduction of the residual spatial structure in the estimated PSU effects (*b*_*u*(*ij*),*k*_’s). Since the PSU effects are estimated at a finer spatial resolution (point scale) than the spatially structured component (region scale), we expect spatial structure may remain in the PSU effects from model (2). Section 4 provides further information.

## 4 Results

The resulting modes of the k-modes algorithm, as seen in Table 1, are almost identical to the initial modes the algorithm was primed with. The sizes of the first three modes, representing “more empowered”, “less empowered”, and “modal empowered” have a fairly even distribution between them and include the majority of the sample of 145,971 with 43,120, 44,325, and 48,821 respondents assigned to each category respectively. The 8848 respondents assigned to category 4 (“autonomous empowered”) and 857 respondents assigned to category 5 (“other”) represent a small minority of the sample, so the remaining analysis focuses on the dominant three. The “modal empowered” category is used as the reference level in the model for the response as described in the previous section.

**Fig. 1:**
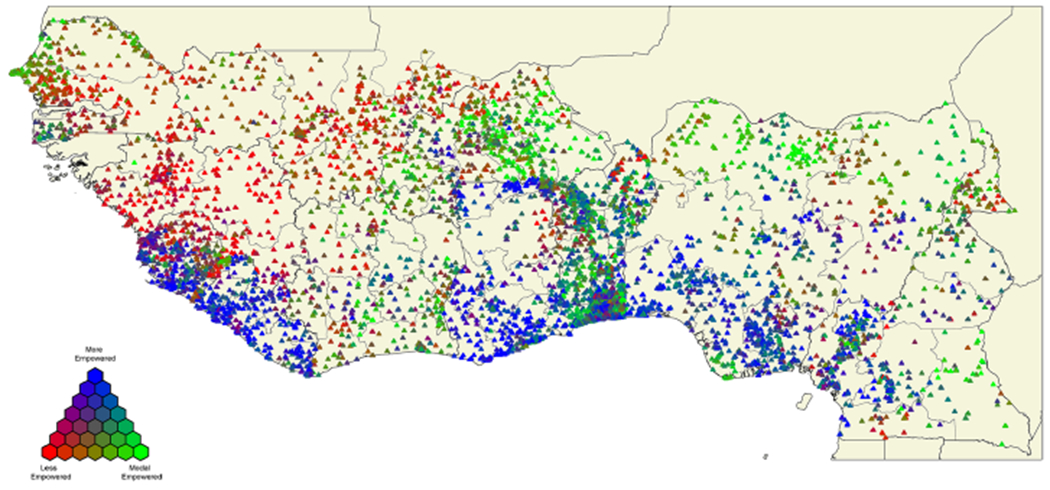
Map of empowerment in West African region with color indicating proportion of women in each empowerment classification by PSU

The three empowerment classifications are depicted on a map of the West African region in Fig. 1.

In this map, a blue point represents a PSU where more respondents are classified as “more empowered”, a red point represents a PSU where more respondents are classified as “less empowered” and a green point represents one where more are classified as “modal empowered”. These colors are blended such that it is easy to visualize the proportion of the population of a PSU that is classified into each empowerment category. Note that data from Niger and The Gambia are not included in this map because latitude–longitude coordinates of respective PSU’s are not available. Using this map, it is evident that there is a clear spatial pattern underlying the empowerment data, and that this pattern transcends administrative boundaries at both a country and subnational level. There is a belt of “more empowered” along the southern coast, namely in Liberia, the southern half of Sierra Leone, throughout Ghana, the coast of Togo and Benin, the southern half of Nigeria, and western Cameroon. The “less empowered” are congregated in the north-western part of the region, notably in Guinea, inland Senegal, and southern Mali. The “modal empowered” are scattered in various concentrations on the map but there are noticeable aggregates of these points within Burkina Faso, Benin, Togo, eastern Cote d’Ivoire, and coastal Senegal. Although clear spatial patterns are apparent on this map, this information alone is not enough to conclude that geography is solely associated with women’s empowerment because it does not take into account the degree to which empowerment-related socioeconomic-demographic characteristics systematically vary by place. Or put differently, the map in Fig. 1 may simply reflect spatial variation in population composition rather than geo-cultural effects.

This motivates our use of the multinomial STAR model which provides estimates of spatial effects while adjusting for age, income, education and type of place of residence (rural/urban). Full Bayesian estimation was performed for both the full and null models (Eqs. 2, 3 respectively), using MCMC in R2BayesX (Umlauf et al., 2015). We used 120,000 MCMC iterations to simulate the posterior distribution, including a burn-in period of 20,000. We used a thinning parameter of 100. Priors were as specified in Sect. 3, except that the prior for spatially dependent parameters {*s_jk_* : *j* = 1, …, *J*, *k* = 1, 2} is only used for the full model. MCMC diagnostics were assessed using the *coda* R library (Plummer et al. 2006) for each element in ***β***_*k*_ for *k* = {1, 2}. Specifically, using the default settings in *coda*:

- The Geweke Diagnostics (Geweke, 1992, using *coda* function geweke.diag) z-score results from comparing the mean for the first 10% of the chain after burn-in, with the last 50% of the chain, all except one (value – 1.98) of the 16 z-scores fell within the desired 95% interval for N(0,1) distribution, namely (−1.96, 1.96). For two other chains run with different seeds, all fell within (−1.96, 1.96).
- The Heidelberger and Welch Diagnostics (Heidelberger & Welch, 1983, using *coda* function heidel.diag) all passed the stationarity test, and the Halfwidth Mean Test.
- Gelman–Rubin Diagnostics (Gelman & Rubin, 1992, based on 3 chains with different seeds, using *coda* function gelman.diag), gives point estimates of the desired value “1”, for the potential scale reduction factor for each element in ***β***_*k*_ for *k* = {1, 2}, with all 97.5% quantiles < 1.02. The multivariate potential scale reduction factor was 1.01 (Brooks & Gelman, 1998). Results reported rounded to 2 decimal values.

For additional details we refer for example to Gelman et al. (2014) and Amaral Turkman et al. (2019).

The results of the multinomial STAR models do provide evidence of a spatial pattern of women’s empowerment while controlling for age, income, education, and type of place of residence. We find that the full model which includes the spatial parameter, *s_jk_*, is preferred to the null model. The deviance information criterion (DIC) value for the full model is lower than that of the null model, supporting this conclusion (Table 3). Spiegelhalter et al. (2002) develop DIC as a penalized Bayesian measure of model adequacy, where the penalty is on model complexity via the effective degrees of freedom. DIC is defined in Table 3 caption. Interestingly, the full model also has lower estimated effective degrees of freedom *p_D_*.

Thus, region explains much of the variation in empowerment classification. Overall the full model shows support that geographic region is spatially associated with estimates of women’s empowerment even when controlling for individual demographic attributes, and that these attributes (age, income, education, rural/urban) though some are notable are unable alone to account for much of the observed variation in women’s empowerment in the region.

**Fig. 2:**
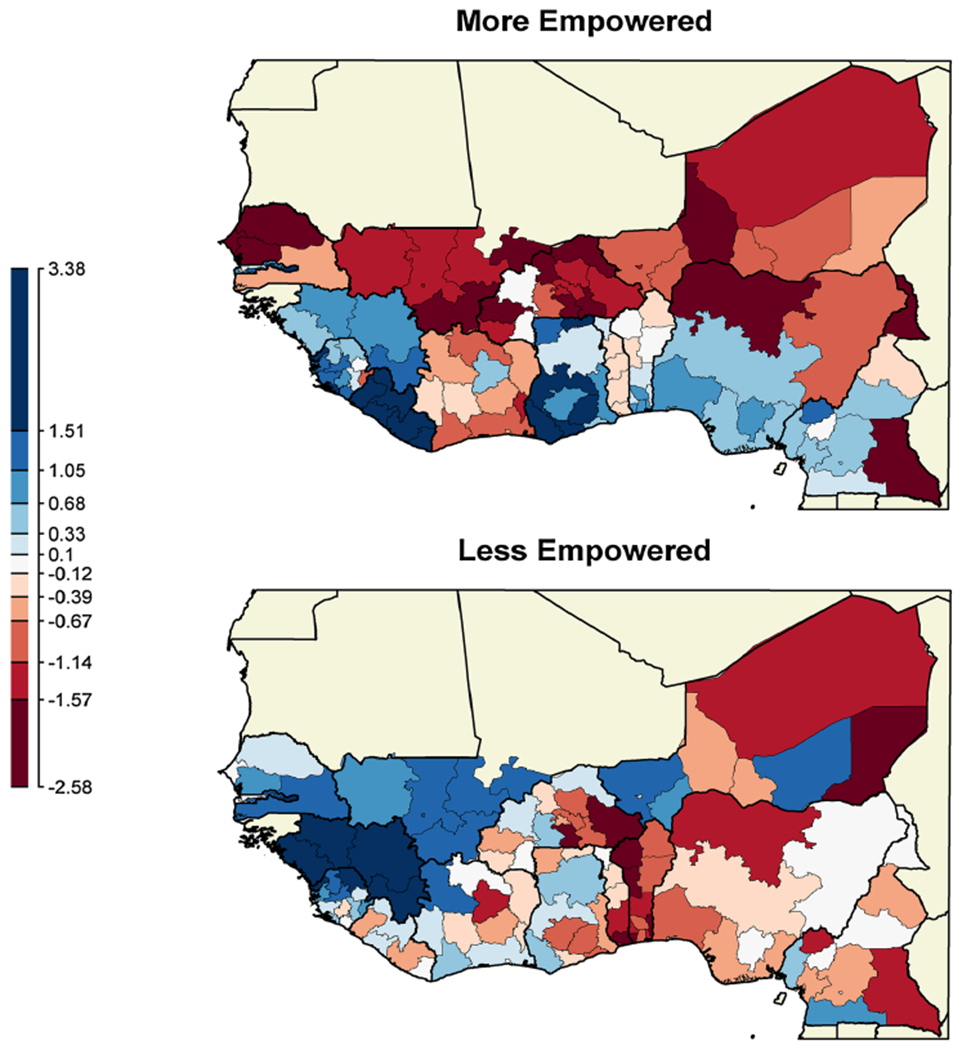
Posterior medians of Markov random field spatial effect estimates for the 120 regions, shown for each *k* ∈ {1, 2} as a map of log of relative odds ratios with reference level “modal empowered”. Zero log-odds are white in color-scale

Estimates of the medians of the posterior distributions of the Markov random field spatial effect *s_jk_* in the full model are depicted as a map, see Fig. 2. Category 3, the “modal empowered” classification, is used as the reference level in the multinomial response. The upper map represents estimates of log odds ratios by first level administrative region of category 1, the “more empowered” versus category 3, “modal empowered”. The lower map represents the same for category 2, “less empowered” versus “modal empowered”.

The spatial patterns on the maps in Fig. 2 resemble those identified in the previous map (Fig. 1) before controlling for education, income, age, and type of place of residence (rural/urban). Blue represents regions with above-zero posterior medians (of log relative odds ratios), thereby indicating that the respective category (upper panel: k = 1; lower panel: k = 2) is estimated to be more prevalent than the reference “model empowered” category in those regions, while controlling for education, income, age, and type of place of residence (rural/urban). Regions in red represent negative posterior medians (of log relative odds ratios), indicating that the respective category (upper panel: k = 1; lower panel: k = 2) is estimated to be less prevalent than the reference “modal empowered” category in those regions, while controlling for education, income, age, and type of place of residence (rural/urban). White areas are estimated to have log relative odds ratios of zero.

Women in the southern half of the map with some exceptions are generally more likely to be more empowered according to our metric. Women in the western half of the map are more likely to be in the less empowered category. Because “more empowered” and “less empowered” are being compared to a third category, “modal empowered”, and not to each other, there is not a perfect complement between the colors of regions when comparing the upper and lower panels. For instance the regions in Guinea are blue in both maps in Fig. 2 suggesting that women are not likely to be in the “modal empowered” category there.

**Fig. 3:**
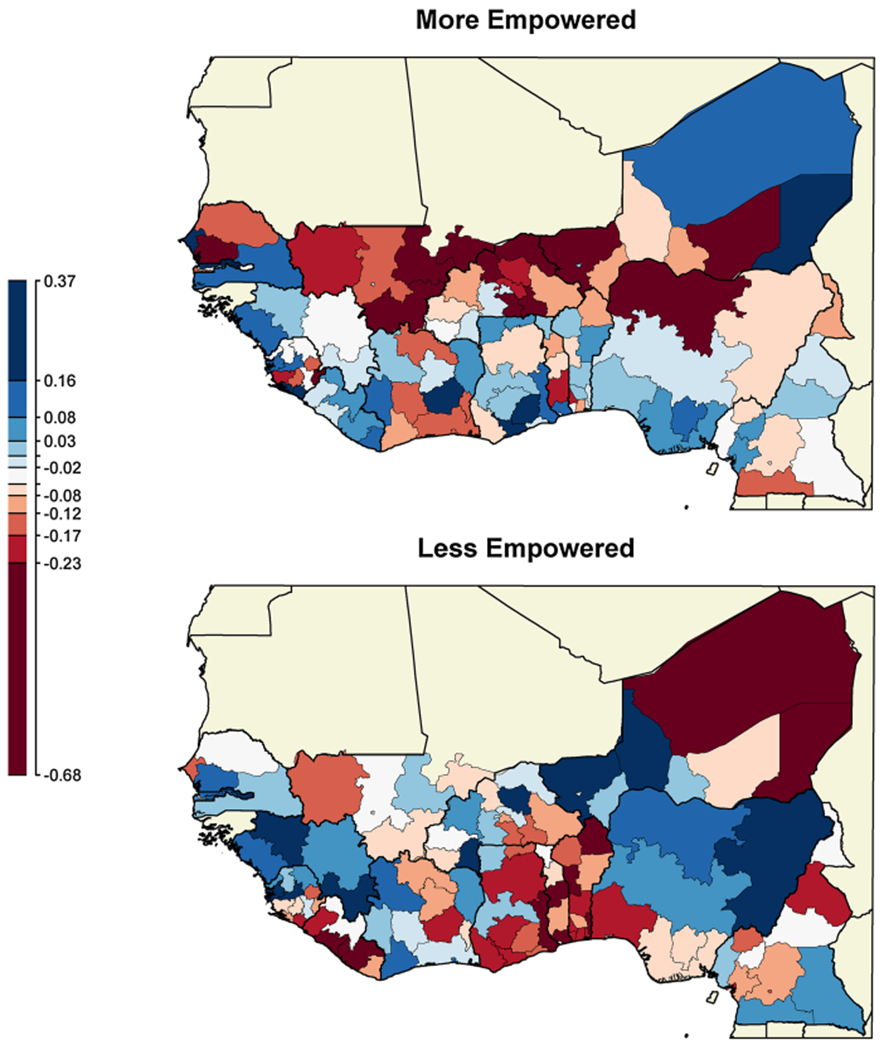
Regional medians of the posterior median of PSU-level effects, from the full model. For each *j* ∈ {1, …, *J* = 120} , all PSUs *u*(*ij*) are located within the *j*th mapped region. There are 7314 total PSUs within study. From our Bayesian Analysis, each PSU (*u*(*ij*)) has a posterior distribution for *b*_*u*(*ij*),*k*_, each with a PSU-specific posterior median, for each *k* ∈ {1, 2}. Within each of the 120 regions mapped, the color corresponds to the median of the PSU-specific posterior medians, where the regional median is taken across all PSU-specific posterior medians of *b*_*u*(*ij*),*k*_ within that region, for each of k = 1 (upper panel) and k = 2 (lower panel). A different color-scale is used compared to Fig. 2

Recall that inclusion of the PSU effects in the model is motivated by the cluster sampling design of the DHS, and allows us to account for intra-cluster sampling correlation in our estimation. In the null model we expect the PSU effects to exhibit strong spatial dependence, and that the spatial dependence will be eliminated or reduced by inclusion of the spatial effect in the full model. We provide a map to summarize the PSU effects at the region scale in Fig. 3. Based on visual inspection, the map appears to still contain some spatial dependence but not as much as in Fig. 2. We can measure the degree of spatial dependence using Moran’s I. The Moran’s I associated with “More Empowered” PSU effect in the null model is 0.52 and that is reduced to 0.18 in the full model. For the “Less Empowered” PSU effects, Moran’s I is reduced from 0.60 (null model) to 0.33 (full model).^1^ Thus the spatial dependence is much reduced, though not eliminated in the full model specification. This is because spatial correlation remains in the PSU effects over short distances, below the resolution of the region lattice.^2^

**Fig. 4:**
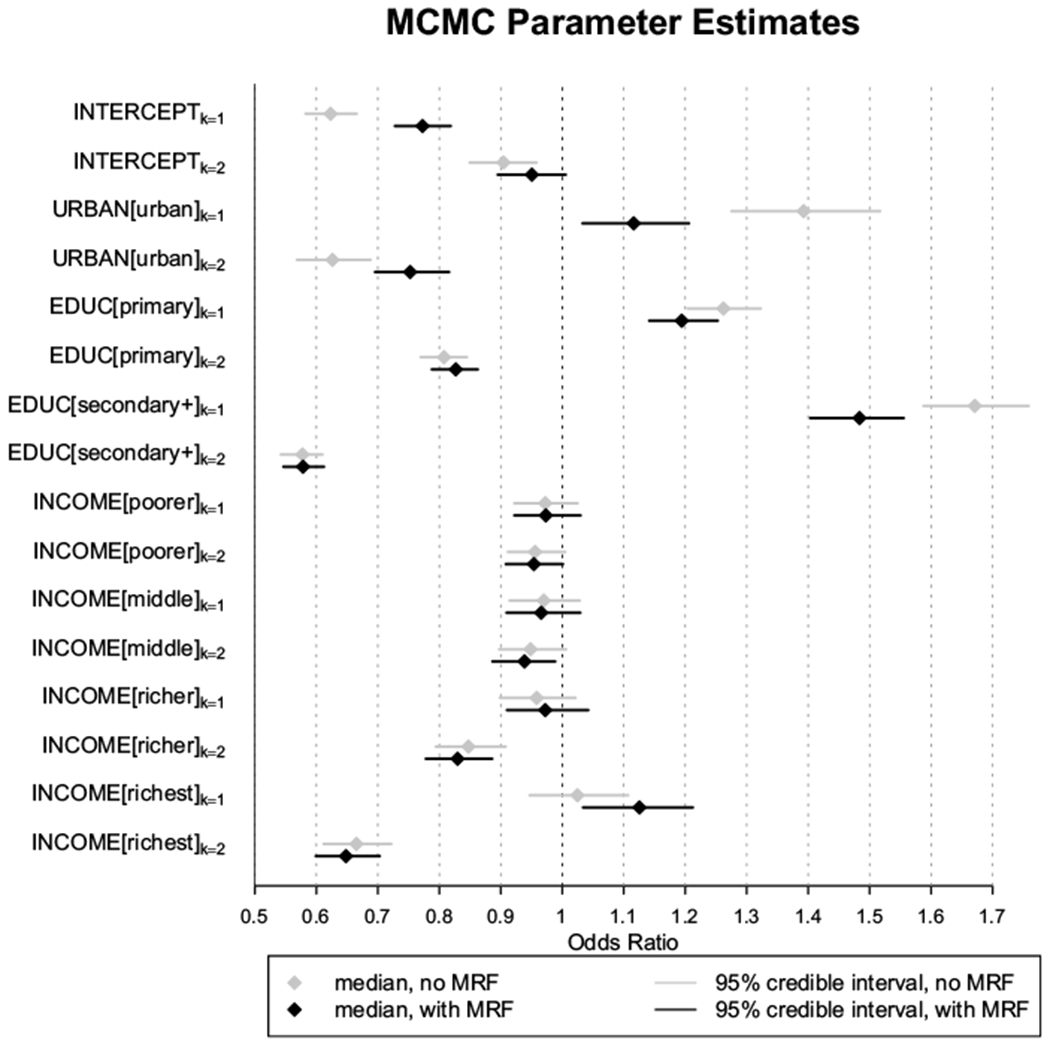
Forest plot of 95% posterior credible intervals for the relative odds ratios {***e***^*β_k_*(*c*)^ : *c* = 1, …, 8} for each *k* ∈ {1, 2}. Results are relative to the reference levels presented in Table 2. For the left hand labels, URBAN is relative to reference “rural” (*c* = 2), EDUC[primary] and EDUC[secondary+] are each relative to reference level “none” (*c* = 3, 4 respectively). Income levels are relative to the reference level “poorest”, corresponding to *c* = 5, 6, 7, 8 in increasing order of income category. Results are presented for each of the null (light grey) and full (black) models. A diamond within each horizontal bar corresponds to the posterior median

Figure 4 presents 95% posterior credible intervals of model parameters ***β***_*k*_ for both the null (light grey) and full models (black) expressed as relative odds ratios ***e***^***β***_*k*_^. Notation of variables relates to terminology in Table 2, for example row label “EDUC[secondary+]_*k*=2_” corresponds to 95% posterior credible interval for ***e***^***β***_*k*=2_(**4**)^ for each of the null (light grey) and full (black) models, relative to the *k* = 3 model reference level and reference levels for the explanatory variables in Table 2. The estimates of the demographic control parameters vary in magnitude and are not all notable. Education is associated with higher estimates of “more empowered” and lower estimates of “less empowered” compared to the reference level, “modal empowered”. The magnitude of these estimates are greater with the higher level of education. Rural/Urban differences are apparent, where urban type of place of residence is associated with being “more empowered” and negatively associated with being “less empowered”, though the size of this effect is smaller when the spatial effect is accounted for. Income appears to be notable only in the highest wealth quintiles and in the expected direction where higher incomes are associated with higher odds of being “more empowered” and lower odds of being “less empowered”. Age is included in the model as a smooth nonlinear predictor. We do not display the age results here, but simply note that age does capture some variation in the relative odds ratios (the credible intervals mostly do not contain 0).

## 5 Discussion

The results of this study support the notion of a geography of women’s empowerment in West Africa, which suggests that women’s empowerment is not only an aspect of individual women but rather equally culturally situated and embedded into place. We find that women’s empowerment, as measured by selected DHS indicators of intrinsic and instrumental agency, varies spatially across the West African region and that relative geographic location is significantly associated with a multidimensional indicator of empowerment when controlling for individual sociodemographic indicators known to be correlated with empowerment. We demonstrate that age, income, education, and type of place of residence, though associated with women’s empowerment, do not explain all the observed variation in empowerment in the region and there persists a significant spatial association while controlling for these socio-demographic indicators. This suggests that how women respond to survey questions about women’s empowerment is related to their social location in place and space in addition to their individual socio-demographic characteristics. This finding, of a broader geographic effect, also suggests that women’s empowerment may be a process that is operating on a larger scale and is more likely due to an acculturation processes of diffusion and social-interaction processes, than merely an individual attribute mediated by economic resources.

Our study builds on the few works analyzing spatial geographies of empowerment (Kazembe, 2020; Kishor & Gupta, 2004; Sarkar et al., 2016). However our study is unique in several key ways. First, the methods employed to construct the multidimensional proxy of empowerment derives from natural groupings in the data. Our finding supports the uncontroversial notion that empowerment is complex and comprised of multiple dimensions. These dimensions may be distinct concepts that could perhaps be measured and analyzed separately. While there are clear natural groupings between what we label as “more empowered” and “less empowered” women, there is also another natural grouping, more sizable than the others, which we refer to as “modal empowered”, that are distinctly different. These are the women who generally disagreed with justifications of wife-beating, supported a woman’s right to refuse sex, but generally said their partners were in charge of household decisions. The fact that this pattern of responses was the most frequent, and more common than that of a consistent “more empowered” or “less empowered” pattern of responses, lends further evidence to support the distinction between multiple independent dimensions of empowerment which do not necessarily correlate. This result is consistent with the literature which describes women’s empowerment as a multidimensional process, where women may become empowered in terms of their intrinsic agency while still disempowered instrumentally.

Second, our study is unique in the way spatial patterns are analyzed across a large region at a finer resolution, using first level administrative regions. Looking within and beyond the country boundaries provides a unique perspective of an African region where national boundaries emerged from a short colonial history and are less meaningfully interpretable than places with a longer historical national identity (Miles, 2015).

Similar to other recent published research exploring regional variation and patterns in women’s empowerment (Kazembe, 2020; Kishor and Gupta 2004), we interpret the spatial effect in our models as indicating residual, spatially-structured variation not captured in the other model covariates. And further, that the spatially-structured variation reflects unmeasured features of the cultural landscape that influence empowerment. To unpack this further, we note that geographers treat space and place as distinct constructs (Cresswell, 2015). Space refers to the purely abstract representation of the locations for a collection of objects and also to connectivity among those objects derived from their relative location. This is reflected in the connectivity matrix we use to encode first-order neighbor relationships among regions (first-level administrative areas) in the countries we use as our West African study area. Place refers to the rich embodiment of culture in a location, which accumulates as a historical process of human habitation, as well as aspects of the natural environment in a location. Women’s empowerment is an element of culture, and we expect it is deeply embedded into and reflects the history of a place; things as complex and long duration as colonial occupation, legal frameworks, educational institutions, or major disruptions due to violent regional/national conflicts are all things that may contribute to the spatial variation in a measure of women’s empowerment. Given the complexity of these elements embedded into culture, and that we do not have measurements of them (and many would be difficult or impossible to measure), we instead rely on allowing them to come through as an aggregate signal in our spatially-structured effect (${\hat{s}}_{jk}$).

We believe the DHS empowerment questions are valid in identifying some dimensions of empowerment in this geographic context and are useful for comparison across geographies. However the work to define, measure, and understand this complex, multidimensional concept is ongoing. Although the sample used in this study was quite large, it was just a small subsample of all the DHS data available about women’s empowerment. Further research could be done to corroborate and expand upon the results found in this study on smaller or other geographic scales. Possible areas of future inquiry could include using clustering algorithms or other multivariate processes to identify patterns in more of the empowerment survey questions on smaller geographic regions or to study country-specific manifestations of empowerment like that by Kazembe (2020). Qualitative and ethnographic research continues to be an important complement to quantitative research in understanding empowerment and validating measures (Randall & Koppenhaver, 2004; Heckert & Fabic, 2013; Olivier de Sardan et al., 2017). Often qualitative data and information from ethnographies is more informative at understanding cultural phenomena like empowerment and should be considered in demographic and development research (Adams, 2016; Randall & Koppenhaver, 2004; Chatterjee & Riley, 2018).

More research is needed to understand geo-cultural specificities and the socio-cultural settings of empowerment in West African populations and their linkages to important demographic processes such as fertility, family planning and women’s health. Considerations of the preconditions and evidence of empowerment at various scales, including not only the well-studied individual characteristics, but also the socio-cultural setting for empowerment in which women are situated and which operates at greater geographic scales would enable a better understanding of the social, economic, and cultural processes and transformations that are underway in the region which function to advance the quality of women’s lives. One policy implication of our principal result is that women’s empowerment is unlikely to change through interventions that only target individual socioeconomic statuses via education or income. International development actors could cater empowerment interventions to smaller geographic regions in unique ways by identifying the local cultural meanings of empowerment and local sources of variation in attitudes and beliefs. Another implication of our results is that, because aspects of women’s empowerment may operate at a level above individuals, empowerment efforts could be effective when operating at these higher levels as well. Identification of empowering—or disempowering—institutions that operate at certain geographic scales, like community, regional, or national levels, could be useful in designing interventions or reforms that address cultural sources of disempowerment and resources for empowerment. Understanding women’s empowerment as a geographic phenomenon—one in which communities and cultures undergo a process that transforms values and practices—provides a framework where more can be done than simply addressing the economic needs of individuals and households.

## Figures and Tables

**Table 1:** Initial and resulting modes of k-modes algorithm

Modes	Empowerment variables used in k-modes clustering		
Initial	Justifications of Violence	Decision-making	Refuse sex

Mode 1	No	With partner	Yes
Mode 2	Yes	Partner	No
Mode 3	No	Partner	Yes
Mode 4	No	Self	Yes
Mode 5	Don’t know	Other	Don’t know

Final	Justifications of violence	Decision-making	Refuse sex

Mode 1	No	With partner	Yes
Mode 2	Yes	Partner	No
Mode 3	No	Partner	Yes
Mode 4	No	{Visit, purchase, health} = self {Spend} = partner	Yes
Mode 5	Don’t know	Partner	No

Variables		Key to DHS empowerment variables used in clustering	

		*Justifications of violence*	
		“In your opinion, is a husband justified in hitting or beating his wife if…”	
Burns		“She burns the food”	
Refuse		“She refuses to have sex with him?”	
Argue		“She argues with him?”	
Neglect		“She neglects the children?”	
Go out		“She goes out without telling him?”	
		*Decision-making*	
Visit		“Who usually makes decisions about visits to your family or relatives?”	
Purchase		“Who usually makes decisions about making major household purchases?”	
Health		“Who usually makes decisions about health care for yourself?”	
Spend		“Who usually decides how your husband’s/partner’s earnings will be used?”	
		*Justified to refuse sex*	
Refuse sex		“Is a wife justified in refusing to have sex with her husband when she knows he has sex with other women?”	

Top panels: Each row corresponds to an initial or final (resulting) cluster mode; Within each row, a single response under one of the three empowerment categories described in the Variable Key (e.g., under the “Justifications of Violence” category), indicates the responses for all variables within that category were the same for this mode; however, within the Decision-making category of the final (resulting) mode 4, different responses occurred, namely “Self” for visit, purchase and health variables, versus “Partner” for the spend category

**Table 2:** The second through eighth elements of categorical covariate vector ***x***_*ij*_, indexed by *c*

Urban/rural (reference “rural”)	Education (reference = “none”)		Income (reference = “poorest”)			
c = 2	c = 3	c = 4	c = 5	c = 6	c = 7	c = 8
1 if Urban	1 if primary	1 if secondary+	1 if poorer	1 if middle	1 if richer	1 if richest
0 if Rural	0 else	0 else	0 else	0 else	0 else	0 else

**Table 3:** Deviance statistics, effective degrees of freedom, and DIC for the null and full multinomial logit models, using the notation of Spiegelhalter et al. (2002) where *θ* represents all parameters in the model

Model	$\bar{D(\theta )}$	$D(\bar{\theta })$	*p_D_*	DIC
	Mean of posterior deviances	Deviance of the posterior means	Effective degrees of freedom	
Null (Eq. 3)	211,825	200,717	11,108	222,933
Full (Eq. 2)	212,296	203,339	8958	221,254

$DIC=D(\bar{\theta })+2p_{D}=\bar{D(\theta )}+p_{D}$ up to rounding: each cell is presented rounded to nearest digit. Lower DIC is preferred

## Footnotes

1 The Moran’s I is based on a first-order row-standardized weight matrix. All effects reported are statistically significant using a one-sided permutation test with 999 replications.

2 We also confirmed this using semi-variograms at the PSU point scale. The total variation captured in the PSU effects is much higher for the null compared to the full model, and the range of the semivariogram is much higher for the null model (roughly 60 km for the null model and 20 km for the full model).
